# Prognostic significance of hemoglobin, albumin, lymphocyte and platelet score in solid tumors: a pooled study

**DOI:** 10.3389/fimmu.2024.1483855

**Published:** 2024-12-18

**Authors:** Jinze Li, Jing Zheng, Puze Wang, Dong Lv

**Affiliations:** ^1^ Department of Urology, People’s Hospital of Deyang City, Chengdu University of Traditional Chinese Medicine, Deyang, China; ^2^ Department of Anesthesia & Operating room, Sichuan Provincial People’s Hospital, School of Medicine, University of Electronic Science and Technology of China, Chengdu, China

**Keywords:** solid tumors, HALP, biological marker, prognosis, survival

## Abstract

**Objective:**

The high hemoglobin, albumin, lymphocyte, and platelet (HALP) score has been reported to be a good prognostic indicator for several malignancies. However, more evidence is needed before it can be introduced into clinical practice. Here, we systematically evaluated the predictive value of HALP for survival outcomes in patients with solid tumors.

**Methods:**

This study was performed according to Preferred Reporting Items for Systematic Reviews and Meta-Analyses (PRISMA) and Assessing the Methodological Quality of Systematic Reviews (AMSTAR) Guidelines. In March 2024, an electronic literature search was performed for articles regarding the prognostic role of HALP in solid tumors. Data from studies with reported risk ratios (HRs) and 95% confidence intervals (CIs) were pooled in a meta-analysis. Study bias was assessed using the QUIPS tool.

**Results:**

Of the 729 articles reviewed, 45 cohorts including data from 17,049 patients with cancer were included in the pooled analysis. The pooled results demonstrated that elevated HALP score was significantly associated with favorable overall survival (HR = 0.60, 95% CI 0.54-0.67, p < 0.01), cancer-specific survival (HR = 0.53, 95% CI 0.44- 0.64, p < 0.01), progression-free survival (HR = 0.62, 95% CI 0.54-0.72, p < 0.01), recurrence-free survival (HR = 0.48, 95% CI 0.30-0.77, p < 0.01), and disease-free survival (HR = 0.72, 95% CI 0.57-0.82, p < 0.01). Subgroup analyses based on various confounding factors further revealed the consistent prognostic impact of HALP on overall survival in patients with solid tumors.

**Conclusions:**

Our findings suggest that high HALP is associated with better survival outcomes in patients. The HALP score is a potential prognostic biomarker in solid tumors, but it needs to be further studied whether it can improve the established prognostic model.

## Introduction

Cancer is a major public health problem worldwide, placing a heavy burden on human health. According to data from the International Agency for Research on Cancer (IARC) in 2020, an estimated 19.3 million new cancer cases and nearly 10 million cancer deaths occurred worldwide (1). Despite significant advances in current cancer treatment, such as the use of immune checkpoint inhibitors and oncogene-targeted drugs, overall cancer-related mortality remains high (2). In addition, cancer treatment varies greatly among individuals, making the prognosis of different individuals significantly different (3). Therefore, there is a need for a reliable biomarker to predict survival in patients with cancer so that therapeutic strategies can be tailored to improve outcomes (4).

Tumor progression and metastasis are not only dependent on the type of tumor cells, but also inflammatory response and nutritional status play important roles in these processes (5, 6). Substantial evidence suggests that parameters reflecting nutritional and inflammatory status, including albumin and hemoglobin levels and lymphocyte and platelet counts, are critical for cancer survival (7–10). The downside of these metrics, however, is that each captures only one aspect of inflammation or nutrition (11). Further studies discovered that a combination of these parameters, including platelet-to-lymphocyte ratio (PLR), neutrophil-to-lymphocyte ratio (NLR), and prognostic nutrition index (PNI), could accurately predict patient outcome more than any single index (12–14). In addition to these well-known markers, a novel inflammatory index combining hemoglobin, albumin, lymphocyte, and platelet (HALP) has been shown to be strongly associated with the prognosis of several malignancies (15–18).

Although a series of studies have attempted to explore the use of HALP as a prognostic marker in human cancer, the results of these findings have been inconsistent (15, 17, 19–22). The advantage of meta-analyses is that they allow pooled effect sizes to be derived from the results of previous studies and thus allow for more robust conclusions to be drawn using data from a large number of patients (23). The purpose of this study was to investigate whether HALP could be a new prognostic indicator for solid tumors using meta-analysis.

## Materials and methods

This meta-analysis was performed following the Preferred Reporting Items for Systematic Reviews and Meta-Analyses (PRISMA) 2020 guideline (24) and A MeaSurement Tool to Assess systematic Reviews 2 (AMSTAR 2) guideline (25). This study was also registered with PROSPERO (CRD42022334548).

### Search strategy

An electronic literature search was conducted on PubMed, Ovid-Embase, Web of Science, and Cochrane Library in March 2024 for articles regarding the prognostic role of HALP in solid tumors. We used the following search terms: “hemoglobin, albumin, lymphocyte, and platelet”, “HALP”, “neoplasm”, “neoplasia”, “cancer”, “tumor”, “carcinoma” and “malignancy”. We also manually searched the literature reference list to further investigate potentially relevant studies. Discrepancies were addressed through discussion or ultimately by third-party adjudication.

### Selection criteria

The criteria for inclusion of studies were as follows: (1) prospective or retrospective clinical studies; (2) studies investigating the association of pretreatment HALP with prognosis in any histologically confirmed solid tumor; (3) patients were adults 18 years of age or older; (4)cut-off values for pre-treatment HALP have been determined and divided into high and low groups; and (5) sufficient data were obtained to assess the hazard ratio (HR) and corresponding 95% confidence interval (CI) between pretreatment HALP and survival outcomes including overall survival (OS), cancer-specific survival (CSS), progression-free survival (PFS), recurrence-free survival (RFS), and/or disease-free survival (DFS). Exclusion criteria were studies categorized as reviews, conference abstracts, letters, and expert opinions. Additionally, unpublished studies, duplicate published studies, studies with insufficient survival data, and studies focusing only on hematological malignancies were excluded.

### Data extraction

Two authors separately collected the following variables from the included studies: first author’s name, year of publication, country, ethnicity, study type, tumor type, tumor stage, treatment strategy, sample size, age of subjects, HALP cut-off value, analysis of survival, survival outcomes (HRs with corresponding 95% CIs for OS, CSS, PFS, RFS, and DFS), and follow-up period. Data were extracted from a multivariate analysis when survival data from a study were analyzed in two ways (univariate and multivariate analyses). Moreover, if relevant data for the article were missing, the corresponding author was contacted. If no response was received or data were not available, the article was excluded.

### Methodological quality

Risk of bias assessment for included studies using the Quality In Prognosis Studies (QUIPS) tool (26). This tool covers six main domains, including study population, study attrition, prognostic factor measurement, outcome measurement, study confounding, and statistical analysis and reporting. Each study was rated as high, moderate, or low risk of bias based on the description in the original study. Two reviewers independently conducted the quality assessment and all disagreements were resolved through discussion or adjudicated by a third party.

### Statistical analyses

We used software R 3.6.3 and Stata 14.0 for statistical analysis. A pooled HR with 95% CI was utilized to assess the association between pre-treatment HALP and survival outcomes. Heterogeneity between studies was estimated using Cochran’s Q test and Higgin’s I^2^ test, and I^2^ > 50% or p < 0.10 demonstrated significant heterogeneity. A random effects model was employed for the combined analysis in this meta-analysis. Moreover, any potential publication bias was evaluated by Begg’s test. We performed subgroup analyses to investigate potential sources of heterogeneity. Meta-regression analysis was conducted to assess the effect of the HALP cutoff value on the HR for OS. Subsequently, sensitivity analyses were also conducted to assess the robustness and reliability of the pooled results. Two-sided p < 0.05 was considered statistically significant.

## Results

### Study characteristics

**Figure 1:**
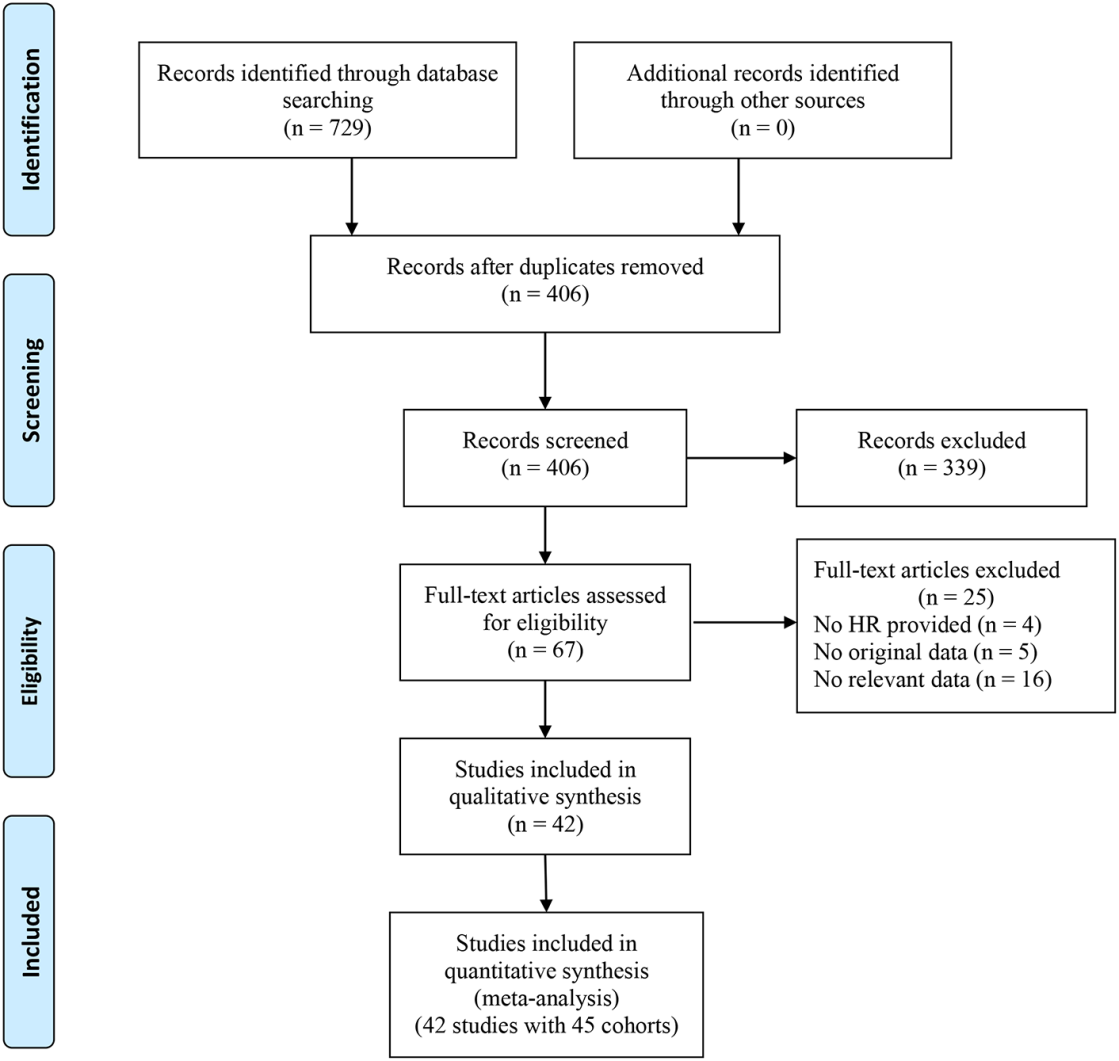
PRISMA flowchart depicting the search strategy used for this study.

The search initially identified 729 articles, leaving 406 articles after eliminating duplicate publications. By reading the titles and abstracts, 339 articles that did not fit the main idea were excluded. The full text of 67 studies was then reviewed, and 25 studies (including 4 studies that did not provide the HR with corresponding 95% CI data, 5 studies with missing survival outcome data, and 16 studies involving patients with non-solid tumors) were excluded. Finally, 42 studies containing 17,049 patients were included in this meta-analysis (11, 15–22, 27–59). The flowchart of the study screening process is presented in **Figure 1**.

Of these 42 studies, three studies had two cohorts (training and validation cohorts) (15, 21, 27), resulting in a total of 45 cohorts included in this meta-analysis. The 29 cohorts were from China (11, 15, 16, 18–21, 27–30, 32–34, 38, 45–49, 53–58), seven from Turkey (17, 31, 35–37, 39, 59), four from Japan (22, 41, 50, 52), three from European and American countries (42, 43, 51), and one study from Thailand (40). In the included cohorts, the most common tumor type was hepatobiliary and pancreatic cancer (n = 8) (21, 30, 31, 48, 50, 57, 58), followed by gastrointestinal cancer (n = 7) (15, 17, 27, 36, 43). Notably, only 4 cohorts were prospectively designed (17, 42, 51, 52), the rest were retrospective (11, 15, 16, 18–22, 27–41, 43–50, 53–59). Of the included cohorts, 31 cohorts underwent curative resection (11, 15, 17–19, 21, 27, 28, 30–36, 38, 41–43, 45, 47–51, 54, 56–58), 9 cohorts received adjuvant therapy (e.g., chemotherapy, radiotherapy, chemoradiotherapy, and immunotherapy) (16, 20, 22, 29, 39, 40, 53, 55, 59), and 2 cohorts received mixed treatment (37, 46). The number of patients included in the individual cohorts ranged from 39 to 1360. The cut-off value of HALP ranged from 0.277 to 56.8. thirty-seven cohorts reported associations between HALP and OS (15–22, 27, 30, 31, 33–37, 39–43, 45, 46, 48–53, 55, 57, 58), 6 cohorts investigated associations between HALP and CSS (11, 27, 32, 42, 46), 8 cohorts examined associations between HALP and PFS (20, 28, 29, 33, 40, 52, 53, 59), 7 cohorts investigated associations between HALP and RFS (38, 42, 47, 48), and 4 cohorts reported associations between HALP and DFS (43, 45, 49, 50, 54, 56, 58). The basic characteristics of the enrolled cohorts are shown in **Table 1**.

**Table 1:** Baseline characteristics of reviewed studies.

Author	Year	Country	Study design	Tumor type	Tumor stage	Treatment strategy	Sample size	Age (years)	HALP Cut-off value	Analysis of survival	Survival outcome	Follow-up (months)
Chen (15) (Training)	2015	China	Retrospective	GC	IA-IV	Curative resection	888	Mean 57.3 ± 11.8	56.8	Multivariate	OS	Median 65.6
Chen (15) (Validation)	2015	China	Retrospective	GC	IA-IV	Curative resection	444	Mean 56.8 ± 11.5	56.8	Multivariate	OS	Median 66
Jiang (26) (Training)	2016	China	Retrospective	CRC	II-III	Curative resection	684	Median 62 (21-92)	26.5	Multivariate	OS, CSS	Median 67
Jiang (26) (Validation)	2016	China	Retrospective	CRC	II-III	Curative resection	136	Median 58 (32-86)	26.5	Multivariate	OS, CSS	Median 68
Cong (20)	2017	China	Retrospective	EC	II-IVA	Chemoradiotherapy	39	Median 60 (45 - 71)	48.34	Multivariate	OS, PFS	Median 27.2
Peng (19)	2018	China	Retrospective	BC	NA	Curative resection	516	Median 66 (57–73)	22.2	Multivariate	OS	Median 37 (20 - 56)
Peng (11)	2018	China	Retrospective	RCC	NA	Curative resection	1360	Median 55 (46 - 65)	31.2	Multivariate	CSS	Median 67 (36 - 74)
Guo (28)	2019	China	Retrospective	Prostate cancer	NA	Curative resection	82	Median 69 (63-73)	32.4	Multivariate	PSA-PFS	17.47
Shen (29)	2019	China	Retrospective	SCLC	NA	Chemotherapy	178	Mean 61.24 ± 9.27	25.8	Multivariate	PFS	NA
Xu (30)	2020	China	Retrospective	Pancreatic cancer	IA-III	Curative resection	582	Median 61 (29 - 82)	44.56	Multivariate	OS	Median 20.9
Yang (16)	2020	China	Retrospective	SCLC	I-IV	Chemotherapy	335	NA	18.6	Multivariate	OS	Median 27.1 (0.5-46.2)
Arikan (31)	2021	Turkey	Retrospective	PAC	NA	Curative resection	129	Mean 64.69	25	Multivariate	OS	NA
Dagmura (17)	2021	Turkey	Prospective	CRC	Mixed	Curative resection	139	Mean 72.82	15.5	Multivariate	OS	NA
Feng (32)	2021	China	Retrospective	EC	I-III	Curative resection	355	Median 59 (36 - 80)	31.8	Multivariate	CSS	Median 34 (4 - 94)
Gao (33)	2021	China	Retrospective	UTUC	NA	Curative resection	533	Mean 66.71 ± 10.4	28.67	Multivariate	OS, PFS	Median 39.6 (21.6 - 65)
Hu (34)	2021	China	Retrospective	EC	I-III	Curative resection	834	Median 60 (55 - 65)	38.8	Multivariate	OS	NA
Sun (21) (Training)	2021	China	Retrospective	BTC	I-III	Curative resection	287	NA	42.68	Multivariate	OS	Median 19 (9 - 37)
Sun (21) (Validation)	2021	China	Retrospective	BTC	I-III	Curative resection	131	NA	42.68	Multivariate	OS	Median 18 (10 - 38)
Topal (35)	2021	Turkey	Retrospective	EC	I-IV	Curative resection	44	27-86	43	Multivariate	OS	NA
Yalav (36)	2021	Turkey	Retrospective	CRC	I-IIIC	Curative resection	279	Mean 61.54	15.7	Multivariate	OS	NA
Zhai (18)	2021	China	Retrospective	NSCLC	IA-IV	Curative resection	238	Mean 62.3 ± 8.4	48	Multivariate	OS	NA
Ekinci (37)	2022	Turkey	Retrospective	RCC	NA	Mixed	123	Median 64 (21 - 81)	0.277	Multivariate	OS	NA
Güç (39)	2022	Turkey	Retrospective	NSCLC	NA	Chemotherapy	401	Mean 63.47 ± 9.75	23.24	Multivariate	OS	Median 18 (1 - 80)
Jiang (38)	2022	China	Retrospective	CC	I-IIA	Curative resection	1054	48.1 ± 9.2	39.5	Multivariate	RFS	Median 53 (9 - 96)
Kurashina (22)	2022	Japan	Retrospective	UC	NA	Immunotherapy	54	70 ± 6.8	30.5	Univariate	OS	NA
Leetanaporn (40)	2022	Thailand	Retrospective	CC	I-IVA	Radiation therapy	1112	Median 52 (44 - 61)	22.2	Multivariate	OS, PFS	Median 2.96
Matsui (41)	2022	Japan	Retrospective	RPS	NA	Curative resection	113	Median 59.7 (17 - 82)	3	Univariate	OS	Median 43.8 (1.8 - 43.8)
Njoku (42)	2022	UK	Prospective	Endometrial cancer	I-IV	Curative resection	439	Median 67 (58 - 74)	24	Multivariate	OS, CSS, RFS	Median 42 (27 – 59)
Ruiz (43)	2022	Mexico	Retrospective	CRC	I-III	Curative resection	640	NA	15	Multivariate	OS, DFS	Median 46.4
Vlatka (44)	2022	Croatia	Retrospective	large B-cell lymphoma	I-IV	Chemotherapy	153	Median 64 (54 - 72)	20.8	Multivariate	OS	Median 40
Wei (45)	2022	China	Retrospective	NSCLC	I-IV	Chemotherapy	362	NA	48.2	Multivariate	OS, DFS	Median 64
Wu (46)	2022	China	Retrospective	Pharyngeal cancer	I-IV	Mixed	319	Mean 57.1 ± 11.5	44	Multivariate	OS, CSS	Median 26.4 (15.6 – 51.6)
Zhao (47)	2022	China	Retrospective	GIST	NA	Curative resection	458	Mean 56.8 ± 12.1	31.5	Multivariate	RFS	Median 56 (4 – 138)
Zhang (48)	2022	China	Retrospective	ICC	I-IV	Curative resection	162	NA	43.6	Univariate	OS, RFS	NA
Fang (49)	2023	China	Retrospective	Oral cavity cancer	I-IV	Curative resection	350	Median 60 (52 - 67)	35.4	Multivariate	OS, DFS	Median 43
Mazzella (51)	2023	Italy	Prospective	NSCLC	I-III	Curative resection	257	NA	32.2	Multivariate	OS	Median 40 (33 – 46)
Nishio (52)	2023	Japan	Prospective	Endometrial cancer	I-IV	Chemotherapy	712	Median 55 (28 -74)	35.52	Multivariate	OS, PFS	NA
Shi (53)	2023	China	Retrospective	EC	II-IVA	Chemoradiotherapy	150	Median 65 (37 - 79)	23.1	Multivariate	OS, PFS	Median 27.5
Toshida (50)	2023	Japan	Retrospective	HCC	NA	Curative resection	332	Median 69 (28 - 87)	45.6	Univariate	OS, DFS	NA
Zhao B (54)	2023	China	Retrospective	NSCLC	IA-IIIA	Curative resection	219	NA	29.31	Multivariate	RFS	Median 24
Zhao R (55)	2023	China	Retrospective	Nasopharynx cancer	III-IV	Chemotherapy	400	Median 48 (40 - 55)	46.61	Multivariate	OS	Median 50 (2-126)
Zhao Z (56)	2023	China	Retrospective	Breast cancer	I-III	Curative resection	411	Median 54.52 (28 - 98)	23.6	Multivariate	RFS	Median 54
Zhou (57)	2023	China	Retrospective	HCC	I-IV	Curative resection	273	53.99 ± 10.74	54.13	Multivariate	OS	56.69 ± 1.25
Huang (58)	2024	China	Retrospective	ICC	NA	Curative resection	227	NA	37.1	Univariate	OS, RFS	Median 15
Mutlu (59)	2024	Turkey	Retrospective	malignant mesothelioma	I-IV	Chemotherapy	115	Median 64 (24 - 83)	25.9	Multivariate	OS, PFS	NA

GC, gastric carcinoma; BC, bladder cancer; BTC, biliary tract cancer; CRC, colorectal cancer; EC, esophageal cancer; SCLC, small cell lung cancer; NSCLC, non-small cell lung cancer; RCC, renal cell carcinoma; PAC, periampullary carcinoma; UC, urothelial carcinoma; UTUC, upper tract urothelial carcinoma; RPS, retroperitoneal soft tissue sarcoma; CC, cervical cancer; GIST, gastrointestinal stromal tumor; ICC, intrahepatic cholangiocarcinoma; HCC, hepatocellular carcinoma; HALP, hemoglobin, albumin, lymphocyte, and platelet; OS, overall survival; CSS, cancer-specific survival; RFS, recurrence-free survival; PFS, progression-free survival; NA, not available.

### Quality of the studies

**Figure 2:**
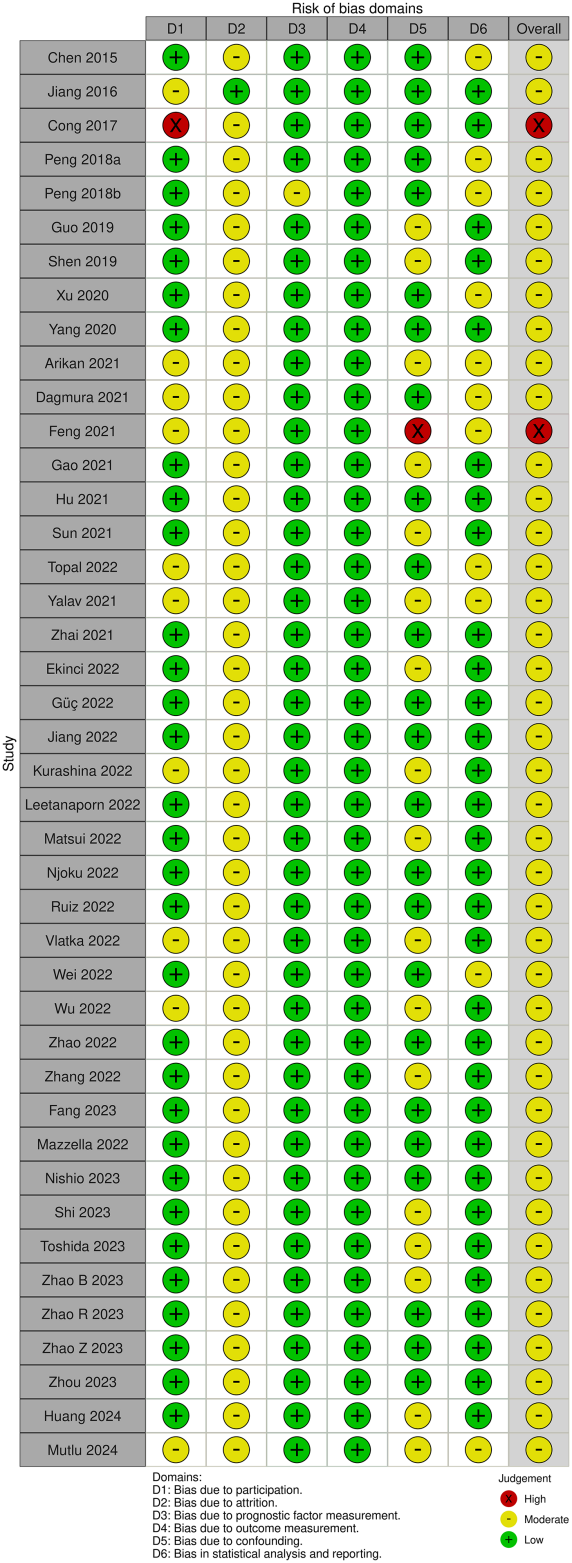
Risk of bias assessment of included studies.

The study quality of each study was assessed using the QUIPS tool. QUIPS domains most commonly evaluated as low risk of bias were the prognostic factor measurement and outcome measurement, while the QUIPS domain most commonly evaluated as a moderate risk of bias was attrition. The majority of studies were judged to be moderate risk of bias and 2 studies were judged to be high risk, as illustrated in **Figure 2**.

### Association of HALP with survival outcomes

#### Overall survival

**Figure 3:**
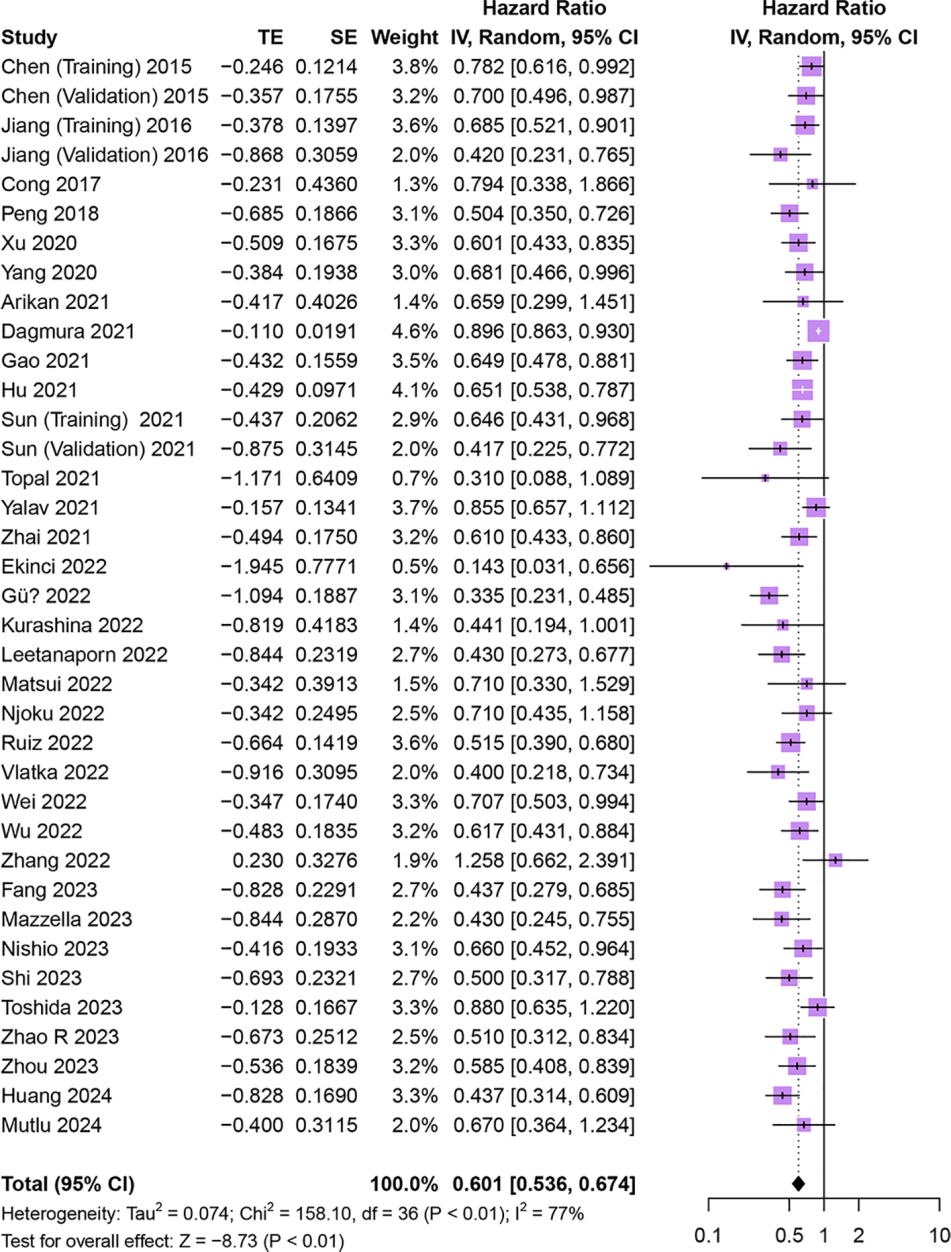
Forest plot showing hazard ratio for overall survival for HALP greater than or less than the cutoff value. HALP, hemoglobin, albumin, lymphocyte and platelet.

Thirty-four studies comprising 37 cohorts investigated the association of HALP with OS in patients with cancer (15–22, 27, 30, 31, 33–37, 39–43, 45, 46, 48–53, 55, 57, 58). The results demonstrated that OS was significantly longer in patients with increased pretreatment HALP (HR = 0.60, 95% CI 0.54-0.67, p < 0.01), with significant heterogeneity among studies (I^2^ = 77%, p < 0.01) (**Figure 3**).

Given the significant heterogeneity between studies, we performed subgroup analyses of OS based on study ethnicity, tumor type, treatment strategy, sample size, study design, analysis mode, cut-off value, and cut-off selection method (**Table 2**). High pre-treatment HALP was found to be consistently associated with better OS regardless of ethnicity, tumor type, treatment strategy, sample size, cut-off value, or cut-off selection method (all p < 0.01). On subgroup analysis stratified by analysis mode, the multivariate analysis subgroup was significantly associated with longer OS (p < 0.01), while the univariate analysis subgroup was not associated with OS (p = 0.08). Furthermore, Meta-regression analysis revealed no significant association between the HALP cutoff value and the HR for OS (p = 0.401, **Supplementary Data Sheet 1**).

**Table 2:** Subgroup analyses of overall survival.

Subgroup	Variable	No. of cohorts	Model	HR (95% CI)	*P*	Heterogeneity	
						I^2^ (%)	*P*
**Ethnic**	Asian	27	Random	0.62 (0.57, 0.67)	< 0.01	20.0	0.18
	Caucasian	10	Random	0.57 (0.43, 0.75)	< 0.01	85.0	< 0.01
**Tumor type**	gastrointestinal cancer	7	Random	0.71 (0.59, 0.86)	< 0.01	78.0	< 0.01
	esophageal cancer	4	Random	0.62 (0.53, 0.74)	< 0.01	0.0	0.46
	hepatobiliary and pancreatic cancer	8	Random	0.63 (0.51, 0.79)	< 0.01	54.0	0.03
	genitourinary cancer	4	Random	0.53 (0.38, 0.73)	< 0.01	35.0	0.20
	lung cancer	5	Random	0.54 (0.40, 0.72)	< 0.01	64.6	0.02
	gynecologic cancer	3	Random	0.59 (0.44, 0.79)	< 0.01	27.0	0.25
	others	6	Random	0.54 (0.44, 0.66)	< 0.01	0.0	0.65
**Treatment strategy**	curative resection	25	Random	0.64 (0.57, 0.72)	< 0.01	76	< 0.01
	adjuvant therapy	10	Random	0.51 (0.43, 0.62)	< 0.01	25.0	0.21
	mixed	2	Random	0.36 (0.09, 1.44)	0.15	70	0.07
**Sample size**	> 300	18	Random	0.61 (0.55, 0.68)	< 0.01	43.0	0.03
	≤ 300	19	Random	0.59 (0.49, 0.71)	< 0.01	75.0	< 0.01
**Analysis mode**	multivariate	32	Random	0.59 (0.52, 0.67)	< 0.01	78.0	< 0.01
	univariate	5	Random	0.69 (0.45, 1.05)	0.08	71.0	< 0.01
**Cut-off value for HALP**	> 26.5	22	Random	0.64 (0.57, 0.71)	< 0.01	39.0	0.03
	≤ 26.5	15	Random	0.57 (0.47, 0.70)	< 0.01	84.0	< 0.01
**Selection of Cut-off value**	ROC analysis	21	Random	0.60 (0.51, 0.71)	< 0.01	81.0	< 0.01
	X-tile software	12	Random	0.59 (0.52, 0.67)	< 0.01	29.0	0.16
	median/mean	3	Random	0.72 (0.54, 0.96)	< 0.01	0.0	0.97
	Cutoff Finder	1	–	0.66 (0.45, 0.96)	0.03	–	–

HALP, hemoglobin, albumin, lymphocyte and platelet; ROC, receiver-operating characteristics; HR, hazard ratio; CI, confidence interval.

#### Cancer-specific survival

**Figure 4:**
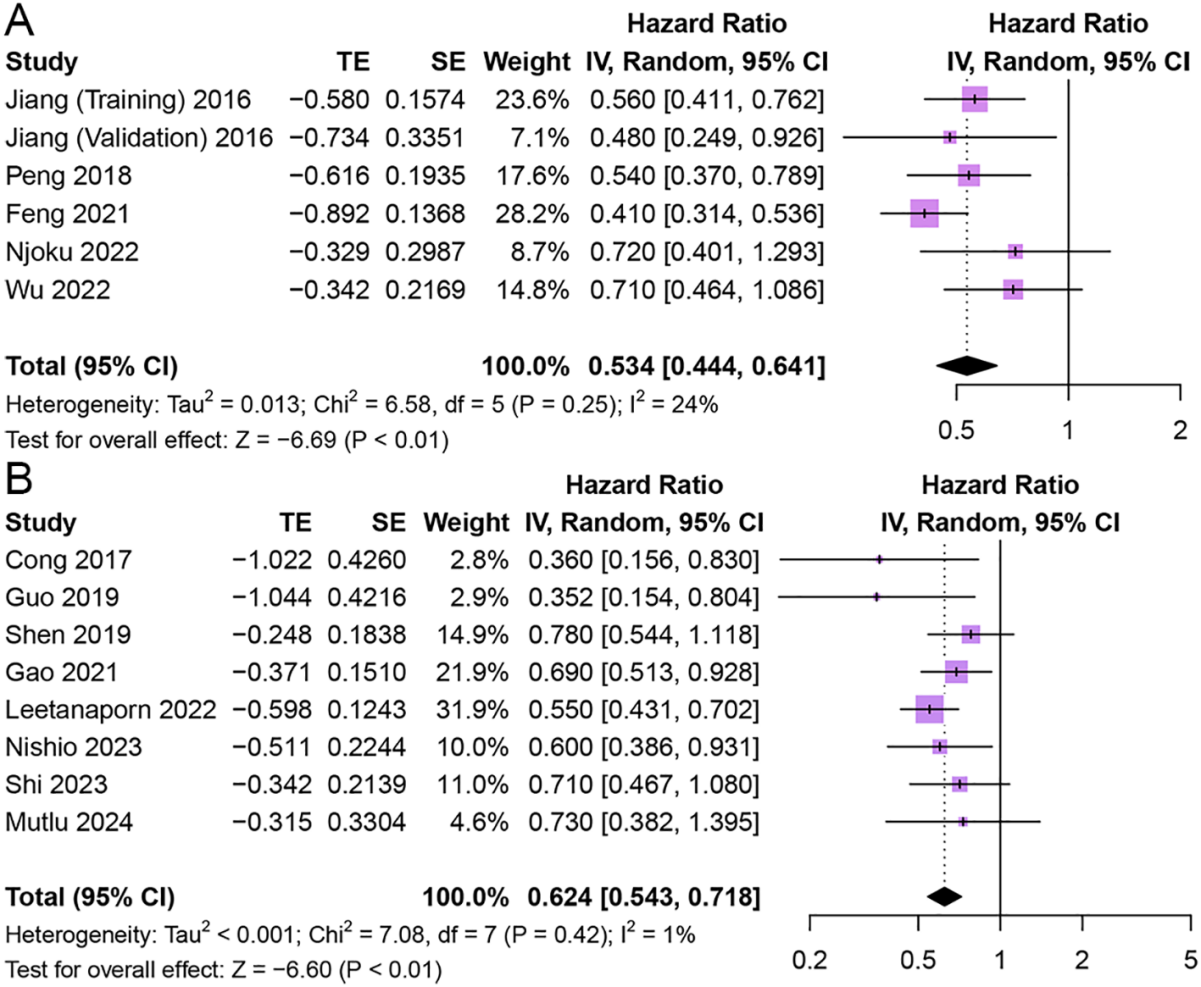
Forest plot showing hazard ratio for cancer-specific survival **(A)** and progression-free survival **(B)** for HALP greater than or less than the cutoff value. HALP, hemoglobin, albumin, lymphocyte and platelet.

Five studies comprising 6 cohorts explored the association of HALP with CSS in patients with cancer (11, 27, 32, 42, 46). The results indicated that higher pretreatment HALP was associated with longer CSS in patients (HR = 0.53, 95% CI 0.44 - 0.64, p < 0.01), and there was low heterogeneity among studies (I^2^ = 24%, p = 0.25) (**Figure 4**).

#### Progression-free survival

Eight studies reported the relationship between HALP and PFS in patients with cancer (20, 28, 29, 33, 40, 52, 53, 59). The results showed that patients with elevated pretreatment HALP had better PFS (HR = 0.62, 95% CI 0.54 - 0.72, p < 0.01), with low heterogeneity between studies (I^2^ = 1%, p = 0.42) (**Figure 4**).

#### Recurrence-free survival

**Figure 5:**
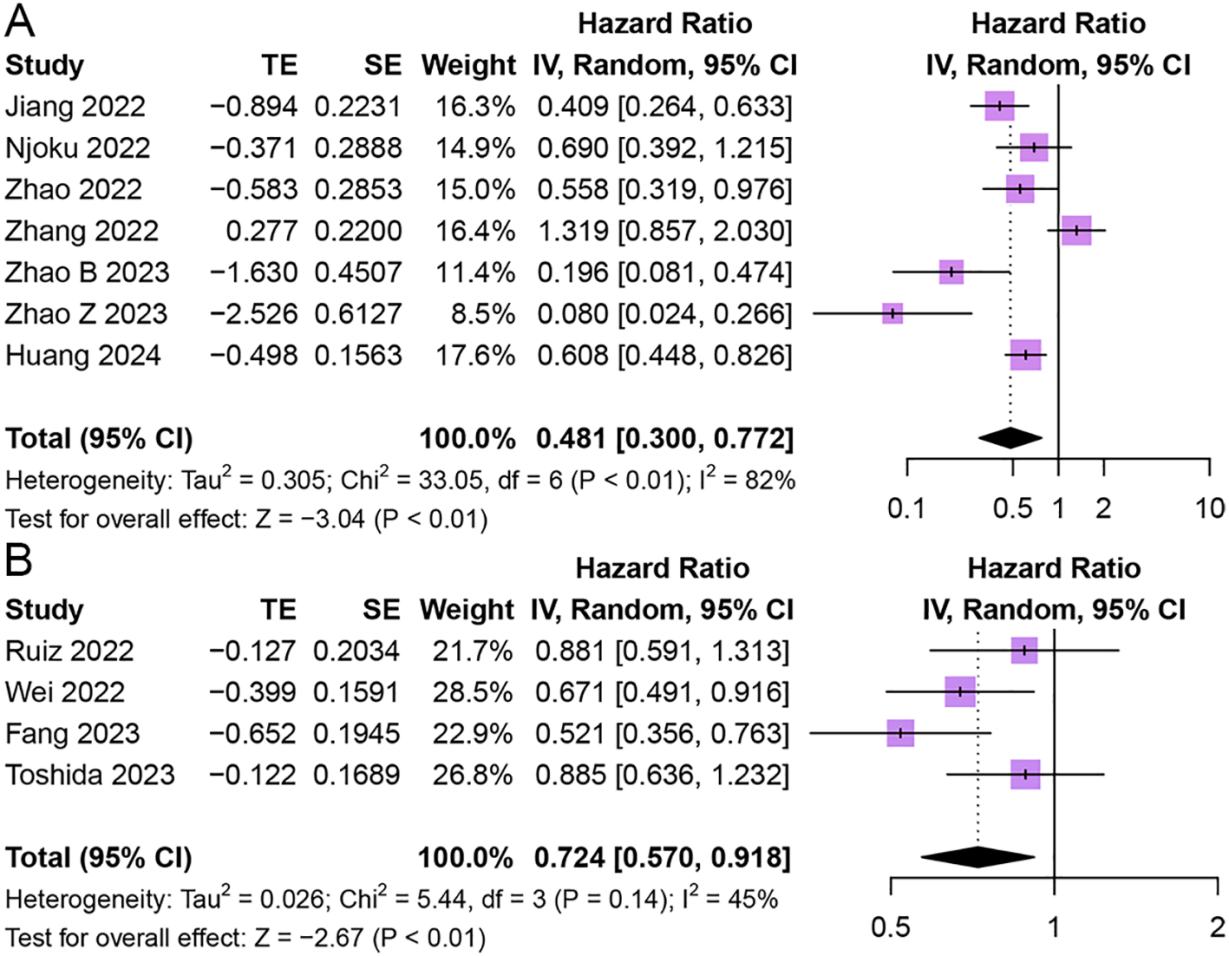
Forest plot showing hazard ratio for recurrence-free survival **(A)** and disease-free survival **(B)** for HALP greater than or less than the cutoff value. HALP, hemoglobin, albumin, lymphocyte and platelet.

Seven studies reported the relationship between HALP and RFS in patients with cancer (43, 45, 49, 50, 54, 56, 58). The results revealed that patients with elevated pretreatment HALP had favorable RFS in patients with solid tumors (HR = 0.48, 95% CI 0.30 - 0.77, p < 0.01), with significant heterogeneity among studies (I^2^ = 82%, p < 0.01) (**Figure 5**).

#### Disease-free survival

Four studies reported the relationship between HALP and DFS in patients with cancer (43, 45, 49, 50). The results demonstrated that patients with elevated pretreatment HALP had better DFS (HR = 0.72, 95% CI 0.57 - 0.92, p < 0.01), with lower significant heterogeneity among studies (I^2^ = 45%, p = 0.14) (**Figure 5**).

### Sensitivity analysis

We performed sensitivity analyses to assess the reliability of pooled HRs for OS (**Supplementary Data Sheet 1**). The exclusion of individual studies had no significant effect on the combined HR, confirming that the results of this meta-analysis are relatively robust and reliable.

### Publication bias

The Begg’s test demonstrated that the results were not statistically significant (OS: p = 0.824), but the Begg’s funnel plots showed asymmetry between the left and right sides, which increases the likelihood of potential publication bias (**Supplementary Data Sheet 1**).

## Discussion

To date, cancer remains the leading cause of death and a significant barrier to increasing life expectancy in all countries of the world (60). Due to the higher cost of cancer management, the establishment of reliable prognostic biomarkers is essential for predicting therapeutic outcomes and determining the patients most likely to benefit from treatment. HALP is a new score based on a combination of inflammatory and nutritional deficiency concepts that was first discovered in 2015 to predict the prognosis of patients with gastric cancer (15). Over the past few years, HALP has been successively used to evaluate survival outcomes in various malignancies. Although a recent systematic review has revealed that low pre-treatment HALP predicts a worse overall prognosis for cancer patients (61), however, there is great heterogeneity in studies investigating HALP in terms of cancer type, outcome, HALP threshold, and population of interest. Here, we conducted an updated meta-analysis based on the available literature to investigate the prognostic impact of HALP. In addition, subgroup analyses were performed to explore the influence of factors such as ethnicity, tumor type, and treatment strategy on the study results.

Evidence from the inclusion of 45 cohorts suggested that an elevated HALP was associated with better OS, CSS, PFS, and DFS in patients with solid tumors. When stratified by ethnicity, disease type, treatment strategy, sample size, and study design higher HALP was consistently an independent factor for favorable OS. Of interest, the included studies reported different HALP cut-off values for different disease types and used different methods to select HALP cut-off values. However, we observed that the prognostic impact of HALP on OS was retained across subgroups. Moreover, in subgroup analyses stratified by analysis mode, HALP scores in the multivariate analysis subgroup were independently associated with OS (**Table 2**). Although no significant difference in OS was observed in the univariate subgroup, it is unlikely to affect the interpretation of our results given the small number of studies included in the analysis. Notably, in this meta-analysis, we included a substantial number of retrospective studies. The subgroup analysis based on study design showed no significant difference between the data from retrospective studies and the overall results (**Table 2**). To some extent, this indicates that data from retrospective studies are consistent with those from other types of studies and did not introduce noticeable bias into the final comprehensive conclusion.

Furthermore, due to the heterogeneity of the studies themselves, we were unable to comprehensively assess the relationship between HALP and age or gender. As age increases, the prognosis of elderly cancer patients is generally worse. However, we observed that almost all studies accounted for patient age when performing multivariate regression or constructing nomograms. Therefore, age does not appear to influence the HALP score. Further research is needed to study HALP scores in healthy populations to accurately evaluate the correlation between HALP and age. Additionally, some studies have reported differences in baseline HALP scores between males and females, but after adjusting for gender, the HALP score remained significant (11, 15, 19, 34). Thus, based on current results, gender does not significantly affect the utility of HALP as a biomarker. In general, a more refined search method and more stringent inclusion criteria were used than in the previous systematic review (61), which dramatically improved the quality and credibility of the study.

The mechanism of the association between high HALP and favorable outcomes in cancer patients remains unclear. One potential mechanism for the prognostic impact of HALP could be the association of high HALP with inflammation and nutrition. Anemia is a well-documented cancer-related phenomenon. In chronic anemia, CD3 T lymphocytes and macrophages release pro-inflammatory cytokines such as IL-6 (62). IL-6 mediates the release of hepcidin from the liver, which inhibits iron absorption and iron release to prevent cancer cells from utilizing iron, thereby reducing erythropoiesis (63). Previous studies also have demonstrated that low hemoglobin levels were associated with adverse clinical outcomes in cancer patients, including impaired quality of life and reduced survival (64, 65). Serum albumin is a reliable indicator for assessing nutritional status and visceral protein function. Studies have reported that in the later stages of the disease, malnutrition and inflammation inhibit albumin synthesis, resulting in lower serum albumin concentrations (66). The reason for this may be due to the production of cytokines, such as IL-6, which regulate albumin production by hepatocytes (67). Furthermore, tumor necrosis factor may increase microvascular permeability, thereby increasing the passage of albumin through capillaries (68, 69). Therefore, mild or no hypoalbuminemia in the early stages of cancer, but a significant decrease in albumin levels as the disease progresses could be a good indicator of cancer prognosis (7).

Abundant evidence indicates that the inflammatory microenvironment is an important component of carcinogenesis. As the basic components of the systemic inflammatory response, platelets and lymphocytes are involved in the continuous inflammation of the tumor microenvironment (70, 71). Platelets have been reported to promote tumor growth and angiogenesis by secreting a mixture of major proangiogenic cytokines in the microcirculation of potentially prothrombotic tumors (72, 73). In addition, platelets also enhance tumor metastasis by covering circulating tumor cells to protect tumor cells from physical factors such as shear stress and host immune responses (72, 74). On the other hand, the importance of lymphocytes has been highlighted in earlier studies. It is an important component of anti-tumor immunity and can inhibit tumor proliferation and migration through cytotoxicity (70). These findings suggest that serum hemoglobin, albumin, and lymphocytes can be considered favorable factors for tumor prognosis, while platelets may be an unfavorable factor.

Over the past decade, energy and resources have been invested in developing biomarkers to help personalize treatment plans for cancer patients. The HALP score combines malnutrition factors (hemoglobin and albumin) with inflammatory response factors (lymphocyte and platelet counts). It may help identify more patients with a poor prognosis than a single index because abnormalities in any single indicator do not truly reflect the patient’s condition. In addition, HALP has even been shown to have the potential to distinguishing between benign and malignant processes (75). Therefore, we reasoned that HALP could serve as a more practical and comprehensive prognostic marker for human cancers, including gastrointestinal, lung, genitourinary tract, gynecological, among others.

### Strengths and limitations

The strength of this study is that it followed international guidelines and a rigorous systematic search and bias assessment protocol were developed in advance. Additionally, this study is the up-to-date systematic review and meta-analysis on this topic and represents the available evidence. Nevertheless, some limitations should be acknowledged. First, this study analyzed aggregated data rather than individual patient data. Second, the majority of the included studies are retrospective, which increases the risk of bias. Future research should prioritize prospective study designs, especially randomized controlled trials, to confirm our conclusions with a higher level of evidence. Third, although stable results were shown in subgroup analyses stratified by treatment strategy, there was a greater heterogeneity in the treatment strategies of patients with different tumors, which could have some potential impact on the study results. Fourth, lymphocyte and platelet counts are non-specific parameters and may be affected by factors such as infection and inflammation (13). Despite most of the included studies have tried to control for these factors, the confounding effects of concurrent inflammatory conditions cannot be completely excluded. Finally, cutoff values for HALP were measured in different ways, and although we did not find a difference between the method of measurement and OS in our subgroup analysis, it is important to establish the optimal HALP cutoff value.

## Conclusions

This study found that an elevated HALP was correlated with better survival in patients with solid tumors, and HALP could be used as a cost-effective prognostic biomarker. The prognostic model based on HALP deserves further investigation.

## Data Availability

The original contributions presented in the study are included in the article/**Supplementary Material**. Further inquiries can be directed to the corresponding authors.
