# Supplementary material for: Prognostic significance of hemoglobin, albumin, lymphocyte and platelet score in solid tumors: a pooled study
Source: Front Immunol. 2024 Dec 18;15:1483855. doi: 10.3389/fimmu.2024.1483855 (PMC11688271; doi:10.3389/fimmu.2024.1483855)
Supplement: Supplementary file 1 [file DataSheet1.docx]

**Supplemental file 1**


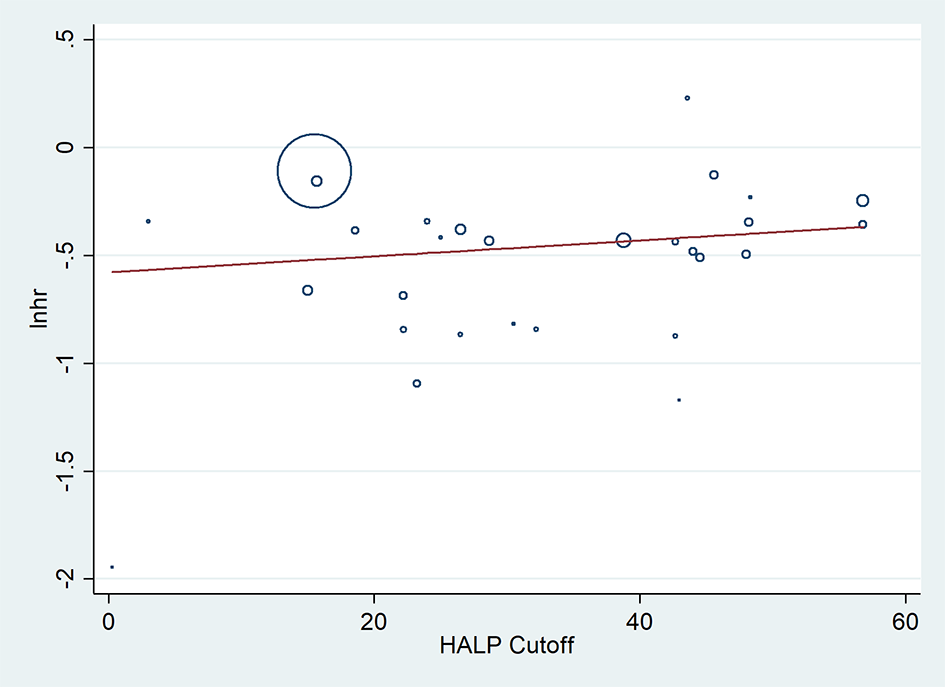


**Figure S1** Meta regression of the association between the cut-off value of HALP and the hazard ratio for overall survival. HALP = hemoglobin, albumin, lymphocyte and platelet.


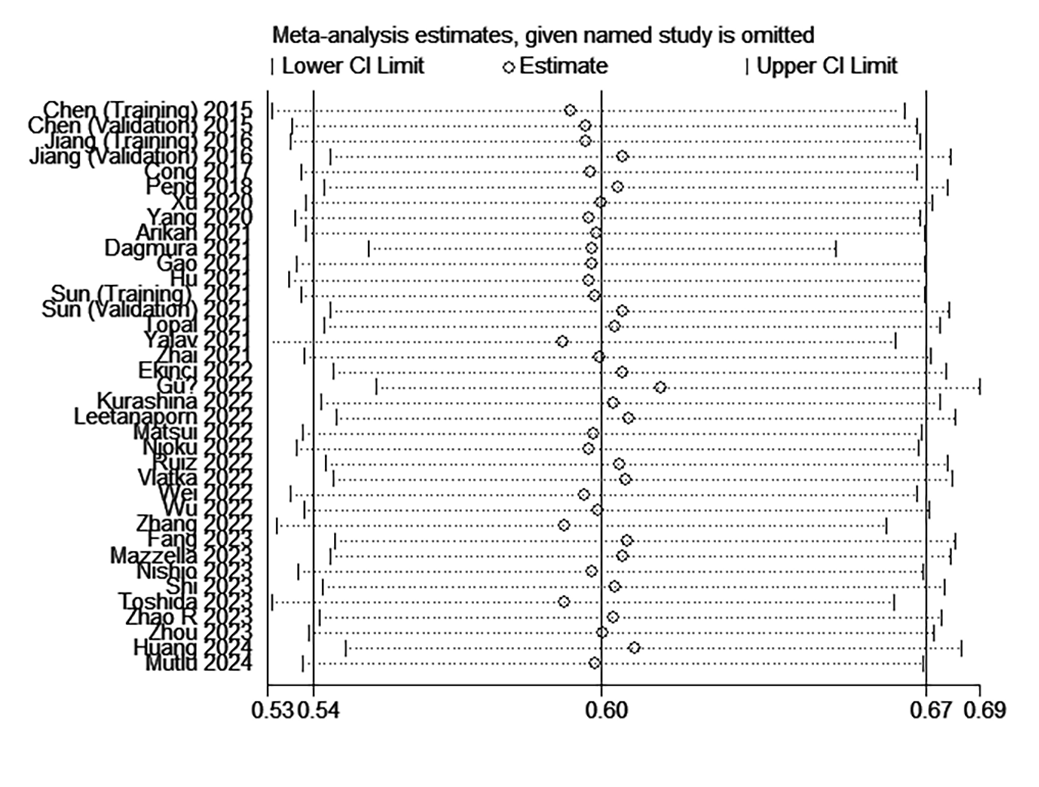


**Figure S2** Sensitivity analysis for overall survival.


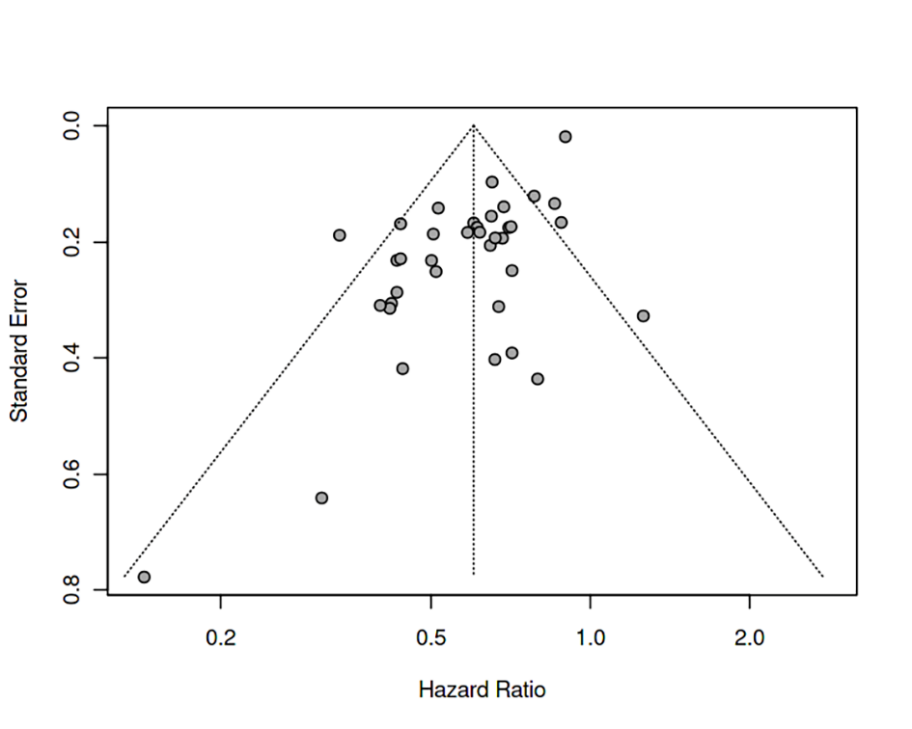


**Figure S3** Funnel plots of the hazard ratio for overall survival.
